# Gut-Brain Axis Cross-Talk and Limbic Disorders as Biological Basis of Secondary TMAU

**DOI:** 10.3390/jpm11020087

**Published:** 2021-01-31

**Authors:** Luigi Donato, Simona Alibrandi, Concetta Scimone, Andrea Castagnetti, Giacomo Rao, Antonina Sidoti, Rosalia D’Angelo

**Affiliations:** 1 Department of Biomedical and Dental Sciences and Morphofunctional Imaging, Division of Medical Biotechnologies and Preventive Medicine, University of Messina, 98125 Messina, Italy; ldonato@unime.it (L.D.); salibrandi@unime.it (S.A.); asidoti@unime.it (A.S.); rdangelo@unime.it (R.D.); 2 Department of Biomolecular Strategies, Genetics and Avant-Garde Therapies, I.E.ME.S.T., 90139 Palermo, Italy; 3 Department of Chemical, Biological, Pharmaceutical and Environmental Sciences, University of Messina, 98125 Messina, Italy; 4 Wellmicro Start Up, Innovative Spin-Off Alma Mater Studiorum Università di Bologna, 40129 Bologna, Italy; andrea.castagnetti@wellmicro.com; 5 Central Health Superintendence, Prevention and Research Division, INAIL, 00144 Rome, Italy; giacomorao@tiscali.it

**Keywords:** TMAU, psychiatric disorders, microbiota

## Abstract

**Background**: Trimethylaminuria (TMAU) is a rare metabolic syndrome characterized by the accumulation and the excretion of trimethylamine (TMA), a volatile diet compound produced by gut microbiota. Gut microbiota alterations are mainly involved in the secondary TMAU, whose patients show also different psychiatric conditions. We hypothesized that the biological activity of several molecules acting as intermediate in TMA metabolic reaction might be at the basis of TMAU psychiatric comorbidities. **Methods**: To corroborate this hypothesis, we performed the analysis of microbiota of both psychiatric suffering secondary TMAU patients and TMAU “mentally ill” controls, comparing the alteration of metabolites produced by their gut bacteria possibly involved in neurotransmission and, in the same time, belonging to biochemical pathways leading to TMA accumulation. **Results**: Microbiota analyses showed that *Clostridiaceae*, *Lachnospiraceae* and *Coriobacteriaceae* alterations represented the bacterial families with highest variations. This results in an excessive release of serotonin and an hyperactivation of the vagus nerve that might determine the widest spectrum of psychiatric disorders shown by affected patients. These metabolites, as short chain fatty acids, lactate and neurotransmitter precursors, are also related to TMA accumulation. **Conclusions**: Knowledge of microbiota-gut-brain axis may become a potential new strategy for improving metabolic diseases and to treat linked psychiatric disorders.

## 1. Introduction

Trimethylaminuria (TMAU) is a metabolic syndrome characterized by the accumulation and the body excretion of trimethylamine (TMA), a compound that can be introduced with diet or synthesized by gut microbiota. TMA is excreted through sweat, breath, urine and other body fluids, determining an unpleasant rotten fish odor. The metabolic and clinical manifestations of TMAU are generally considered benign, as there is no associated organ dysfunction. Such evaluation, as well as the evidence that the condition is frequently unrecognized by clinicians, can have important consequences on the delayed or missed diagnosis [1].

The incidence of heterozygous carriers for this pathology ranges from 0.5 to 11 percent depending on the ethnicity examined [2]. Today, at least two different types of TMAU are differently recognized: The Type 1, caused by a deficit of the Flavin-containing monooxygenase 3 (FMO3) enzyme, and the secondary TMAU, determined by other-than-genetics factors, such as gut microbiota alterations [3].

The *FMO3* gene belongs to the family of FMO genes, and encodes for a transmembrane protein localized to the endoplasmic reticulum of several tissues, particularly in the liver [4]. The FMO3 triggers the NADPH-dependent oxygenation of various sulfur-, nitrogen- and phosphorous-containing xenobiotics such as therapeutic drugs, pesticides, and dietary compounds like TMA and tyramine. In particular, the FMO3 catalyzes the N-oxygenation of TMA, synthesized after the ingestion of choline, lecithin and L-carnitine rich foods, in trimethylamine-N-oxide (TMAO), which is an in-odorous molecule [5]. Consequently, when the pathological condition is suspected or known to occur in a family, the genetic test of the *FMO3* gene can be helpful in identifying members who present the disorder or carry causative variant. Most of TMAU cases are indeed inherited with an autosomal recessive pattern [6]. 

Although *FMO3* mutations occur in most of TMAU patients, an increasing number of cases are caused by other factors [7]. A fish-like body odor could result from an excessive intake of certain proteins with diet or from increase of specific bacteria families in the digestive system. Among secondary TMAU causes, indeed, the dysbiosis of the gut microbiota is the most frequent. The normal flora present in certain body districts could play a key role in determining the age of onset and, above all, the phenotype, particularly variable from patient to patient. The intestinal microbiota is involved in the conversion of choline, carnitine, lecithin - present in some foods - into derivatives of TMA, which are then absorbed by the intestinal mucosa. Several species of commensal microorganisms characterized by a more active metabolism, as well as an overexpressed microbiota, could determine a greater accumulation of TMA, thus causing a more serious phenotype, and/or an early clinical onset [8].

The TMAU pathological condition is uncommon in the society [8], and due to the fish odor, affected people are often marginalized. This social impact is commonly considered the first cause of the psychiatric conditions as depression, anxiety, behavior disorders that affect people with TMAU. The patients feel shame and embarrassment, fail to maintain relationships, avoid contact with people who comment on their condition and are obsessive about masking the odor with hygiene products and even smoking. Moreover, the malodorous aspect can have serious and destructive effects also on schooling, personal life, career and relationships, resulting in social isolation, low self-esteem and suicide. Several evidences suggest that biological and physiopathological cellular alterations could link TMAU with nervous disturbs [9].

From a careful analysis of the structure of TMA, it is possible to observe a strong structural analogy with homocysteine and, therefore, it is likely to hypothesize that, just as in homocystinemia, at the basis of most of the pathological conditions associated with trimethylaminuria there is an excess of TMA derivatives in the blood responsible for excitotoxicity, oxidative stress, inflammatory phenomena and endothelial dysfunction. Oxidative stress and inflammation are both responsible for endothelial dysfunction implying, at the brain level, the alteration of the endothelial junctions and, therefore, an increase of the blood brain barrier (BBB) permeability. Such impairment could determine, in the long run, a relevant excitotoxicity, responsible for neuronal degeneration [10].

The molecular basis of the physiopathological excitotoxic mechanism is a strong structural analogy between homocysteine and glutamate, one of the most important excitatory neurotransmitters in the brain. Thus, the excess of homocysteine is responsible for a prolonged and excessive activation of N-Methyl-d-aspartate (NMDA), post-synaptic glutaminergic receptors. Its activation is accompanied by the influx of Ca^2+^ resulting in molecular damage, loss of mitochondrial membrane potential and increased oxidative stress [11,12], release of metabolites in to the extracellular space. Based on structural homology between homocysteine and TMA, a similar excitotoxic mechanism might be hypothesized to explain psychiatric behavior in TMAU patients. However, given the poor understanding of the mechanism underlying this rare metabolic disorder, it is still unknown if the psychiatric involvement is a cause, or conversely, a consequence of TMA altered metabolism. Several elements, indeed, let us hypothesize that the biological activity of several molecules acting as intermediate in TMA metabolic reactions might be at the basis of TMAU psychiatric comorbidities. In order to corroborate this hypothesis, we performed the analysis of microbiota of both psychiatric suffering secondary TMAU patients and TMAU “mentally ill” controls, comparing the alteration of their bacterial produced metabolites possibly involved in neurotransmission and, in the same time, belonging to biochemical pathways leading to TMA accumulation.

## 2. Materials and Methods

### 2.1. Subjects

Microbiota comparative analysis of 7 secondary TMAU affected patients with behavior disorders (from now formerly indicated as “case”) and 5 demographically TMAU matched control subjects without cerebral functional impairments (called “controls”), all between the ages of 20 and 72 years, participated in this work. The secondary TMAU pathological condition was assessed by negativity of genetic test on *FMO3* gene and with urinary TMA dosage. The behavioral alterations were clinical diagnosed, basing on patients’ anamneses. Control participants were recruited after clinical assessment of healthy mental state using the Mini-International Neuropsychiatric Interview, excluding from the analysis subjects with past or present diagnosis of a major neuropsychiatric illnesses [13]. We established the nearest matching neighbors evaluating sex, age, race, BMI category (obese vs. not obese), and history of antibiotic use (in the past year) to control for clinical factors and known major drivers of microbiome changes [14] that could act as confounding factors. More details about subjects are available in Table 1. All participants provided written informed consent.

### 2.2. DNA Extraction and Sequencing

Total genomic DNA was extracted from fecal specimens using the QIAamp PowerFecal DNA kit (Qiagen, Hilden, Germany), following the protocol provided by the manufacturer. Then the DNA was quantified by spectrophotometric reading of the absorbance at 260 nm by the QIAExpert (Qiagen, Hilden, Germany) and the quality was verified by electrophoretic run on the QIAdvanced (Qiagen, Hilden, Germany). The V3 and V4 regions of the 16S rRNA coding gene were amplified with primer SD-Bact-0341-bS-17/SD-Bact-0785-aA-21 [15] in 25 µL of final volume of PCR mix consisting of 2x PCRBIO Taq Mix (PCR biosystem, London, UK) and 2.5 µL of DNA (5 ng/µL). The thermal cycle was set with an initial denaturation at 95 °C for 3 min, 25 denaturation cycles at 95 °C for 30 s, annealing at 55 °C for 30 s, extension to 72 °C for 30 s and a final step extension at 72 °C for 5 min. The 460 bp amplicons were purified using a magnetic bead system (Agencourt AMPure XP; Beckman Coulter, Brea, CA, United States) and the libraries prepared using the Nextera V2 indexes (Illumina, San Diego, CA, United States). The samples were, then, normalized to 4 nM, denatured and diluted to 5 pM before being loaded onto the MiSeq sequencer (Illumina, San Diego, CA, USA).

Raw sequences were processed using a pipeline that combines PANDAseq [16] and QIIME [17]. The high-quality reads were grouped into Operational Taxonomic Units (OTUs) using UCLUST [18] with a 97% similarity threshold. Taxonomy was assigned using the Greengenes database (May 2019).

### 2.3. Statistical Analysis

The whole statistical analyses were executed using IBM SPSS 26.0 software (https://www.ibm.com/analytics/us/en/technology/spss/). Bonferroni corrected *p*-values < 0.05 were considered as statistically significant. Significant differences in alpha diversity were elaborated with QIIME by pairwise non-parametric *t*-test with 999 permutations. Significant differences in beta diversity were computed with QIIME by PERMANOVA, and permDISP permitted us to check for significant differences in dispersion [19,20]. Taxonomic comparisons were performed by Analysis of Composition of Microbiomes (ANCOM), which exploits compositional log-ratios to identify statistically significant taxa [21]. Canonical Correspondence Analysis (CCA) [22] was implemented with the R package “vegan”, and its significance (consisting of the variables sex, age and TMAU affected or not) was tested with ANOVA and step-wise analysis, and corrected by Bonferroni post-hoc method.

### 2.4. Neurotransmission Pathway Analysis of Gut-Brain Axis

Starting by obtaining OUT relative abundance, we hypothesized the possible role of each altered microbial species in relation to neural alterations. Therefore, we deeply explored literature and MetAboliC pAthways DAtabase for Microbial taxonomic groups (MACADAM), a user-friendly database rich of statistics about metabolic pathways at a given microbial taxonomic position [23]. For each prokaryotic complete genome retrieved from RefSeq, MACADAM creates a pathway genome database (PGDB) exploiting Pathway Tools software built on MetaCyc data which includes metabolic pathways, associated metabolites, enzymes and reactions. Too guarantee the highest quality of the genome functional annotation data, MACADAM also includes Functional Annotation of Prokaryotic Taxa (FAPROTAX), a manually curated functional annotation database, MicroCyc, a manually curated collection of PGDBs, and the IJSEM phenotypic database.

## 3. Results

### 3.1. Microbiota of Neuro-Disordered TMAU Patients Revealed Huge Differences in Composition and Relative Abundances If Compared with “Brain-Healthy” TMAU Affected Individuals 

Microbiota comparative analysis of TMAU cases versus controls highlighted very interesting differences, regarding both bacterial family heterogeneity and concentration (Figure S1). Microbiotas of cases showed a prevalent over-abundance of bacteria (10 families), with *Clostridiaceae* reaching the highest values in 4 cases, and *Enterococcaceae* in 2. The lowest abundance, instead, was highlighted by *Lachnospiraceae* (3 cases) and *Coriobacteriaceae*, reduced in two cases. The most altered family both in cases and controls was the just cited *Lachnospiraceae* which, however, showed an opposite trend, reaching the highest relative abundance in controls (about 72.24%), and the lowest in cases (from 1.86% to 3.78%). The absolute lowest abundances were achieved by *Streptococcaceae* and *Coriobacteriaceae* in cases (0.01%), and by *Enterobacteriaceae* and *Sutterellaceae* in controls (0.01%). Among cases, the n° 6 highlighted the highest number of bacterial family with expression alterations (*Enterococcaceae* = 0.68%; *Erysipelotrichaceae* = 3.9%; *Rikenellaceae* = 6.95%; *Streptococcaceae* = 2.62%; *Lachnospiraceae* = 3.78%; *Coriobacteriaceae* = 6.5%), while the control showing the most differentially expressed bacterial family was the 4c (*Enterobacteriaceae* = 2.8%; *Oxalobacteraceae* = 0.08%; *Erysipelotrichaceae* = 3.8%; *Rikenellaceae* = 6.78%; *Veilloneaceae* = 0.48%; *Roseburia* = 1%). Detailed list of differentially represented bacterial families and genera in case and controls is available in Table 2.

### 3.2. Altered Bacterial Families of Neuro-Disordered TMAU Patients’ Microbiomes Produce Neurotransmitters and/or a Wide Range of Metabolites Involved in Their Biochemical Pathways

All identified microbial families share a very interesting feature, consisting in the common production of a very heterogeneous and rich group of metabolites involved in neurotransmitter biosynthesis and degradation, as well as in their biochemical pathways required to the correct physiology of chemical synapses. Enterobacteriaceae are able to directly synthetize dopamine, norepinephrine and serotonin, while Roseburia, Clostridiaceae and Veilloneaceae could produce the highest number of different metabolites (acetate, lactate, butyrate, propionate, succinate and valeriate). A complete list of all metabolites produced by considered bacteria, involved in nervous physiology, is available in Table 3. 

Linking the alterations of microbiota families to each metabolite produced, a possible complex scenario emerged from analysis of biochemical patterns. The short-chain fatty acids (SCFAs) resulted the most altered molecules in both case and controls, even if with different trends, with the propionate more differentially produced in cases. Tryptophan and GABA, instead, showed different levels only in controls, in which resulted down-represented (Table 4). 

### 3.3. Pathway Analysis of Differential Abundances of Bacterial Families Suggested a Possible Biochemical Link between Microbiota Produced Metabolites, TMA Biosynthesis and Mood/Behavioral Disorders

**Figure 1:**
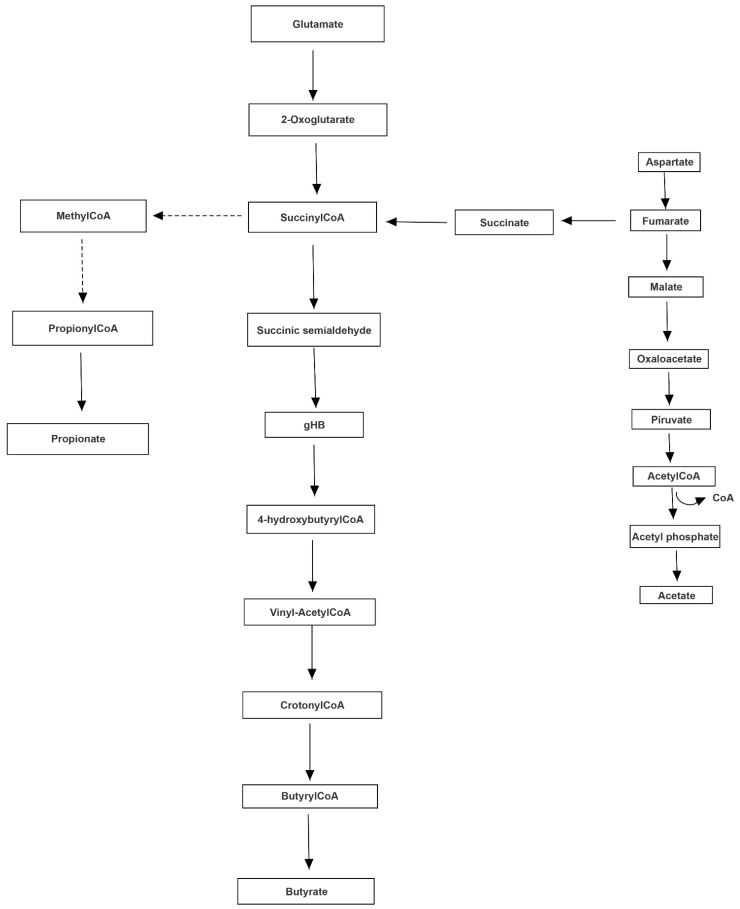
Mixed acid fermentation involving microbiota bacteria. The metabolic way of mixed acid fermentation could produce short chain fatty acids, able to determine an excess of serotonin.

**Figure 2:**
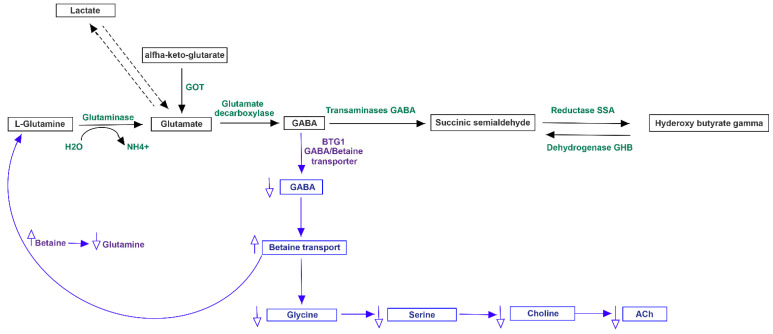
Metabolism of glutamate and GABA linked to ACh. The complex pathway, showing also the involvement of lactate, could play a relevant role in regulation of betaine, a precursor of TMA.

Both MACADAM and literature analyses showed a very interesting network involving main metabolites produced by microbiota, TMA precursors and neurophysiological pathway [24]. Differential production levels of SCFAs (acetate, propionate and butyrate, also resulted from mixed acid fermentation, Figure 1), together with lactate and α-ketoglutarate play a fundamental role into biogenesis of glutamate and GABA, whose concentration could interfere with betaine transport, determining a possible accumulation of TMA [25] (Figure 2).

**Figure 3:**
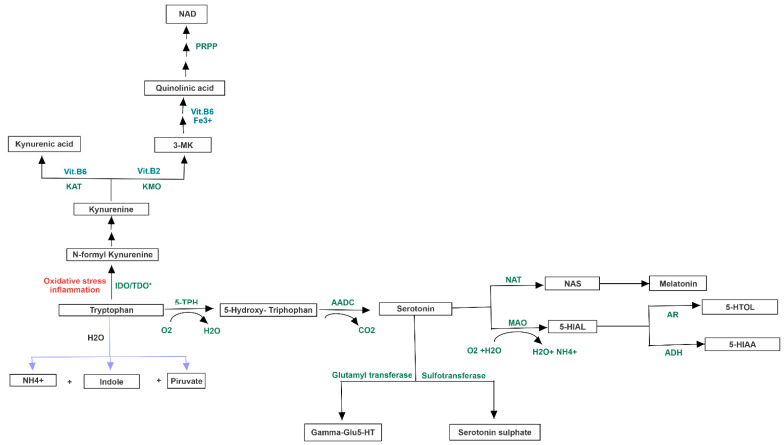
Serotonin metabolism and its “shunt” following oxidative stress and inflammation. Serotonin, produced from tryptophan, could be converted in melatonin. In condition of oxidative stress and inflammation, the amino acid shifts to kynurenine and quinolinic acid pathway, exerting neurotoxic effects.

The same biological process could be activated by serotonin, produced from amino acid tryptophan, and whose release is induced by high levels of lactate [26]. Furthermore, the biosynthesis of serotonin is strictly connected to melatonin one, whose involvement in circadian rhythms such as sleep-wake cycle is well known. Interestingly, in condition of elevated oxidative stress and inflammation, the tryptophan could shift from serotonin biosynthesis to quinolinic acid one, a neurotoxic byproduct able to induce depression (Figure 3). 

**Figure 4:**
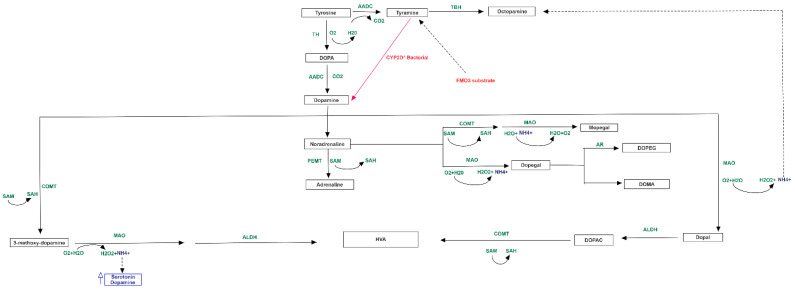
Metabolism of catecholamine and link to serotonin. The scheme also shows that tyramine, produced from tyrosine, is a substrate of FMO3.

Catecholamine metabolism resulted also involved in TMA accumulation. The concentration of norepinephrine, synthetized by dopamine, could regulate the activity of Phosphatidylethanolamine N-methyltransferase (PEMT) enzyme, which is also able to metabolize the phosphatidylethanolamine into phosphatidylcholine [27], which then could be converted to choline, with final increase of TMA levels (Figure 4). 

**Figure 5:**
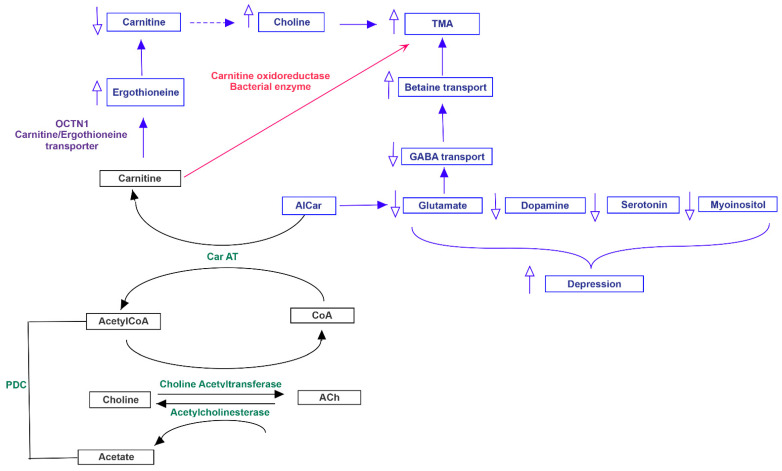
Acetylcholine and carnitine metabolism could influence TMA accumulation and behavioral phenotype. Both carnitine and acetylcholine could alter choline and acetyl-carnitine biosynthesis, determining an accumulation of TMA. In the same time, the acetyl-carnitine could influence the release of main neurotransmitters, determining important behavioral alterations.

The choline quantity could be also raised by acetylcholine, which could also play an important role in carnitine biosynthesis, that could be converted to TMA by bacterial carnitine oxidoreductase (Figure 5). 

**Figure 6:**
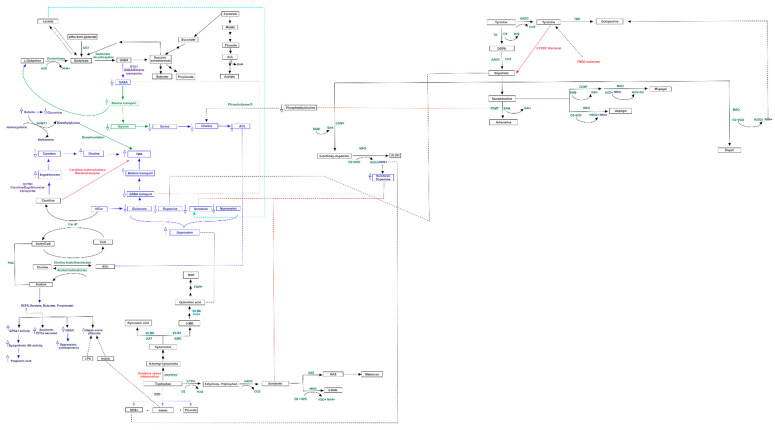
Detailed diagram of biochemical pathways linking neurotransmitter and TMA metabolisms. The figure represents how neurotransmitter and TMA pathways might be correlated. Dashed lines represent indirect and candidate relationships. Empty arrows indicate over- or -down-expression of adjacent metabolite [28].

Fluctuation of described neurotransmitters could lead to vagus activation/deactivation and limbic deregulation, with behavioral and mood disturbs, like one evidenced by cases in exam. A detailed scheme of all evaluated biochemical pathways linking neurotransmitter and TMA metabolisms is represented in Figure 6.

## 4. Discussion

Alterations of microbiome is at the basis of an increasing number of metabolic disorders [29]. Recently, it has been highlighted that the gut microbiome is also linked to brain physiopathology [30]. Regarding this, the gut microbiome-brain axis is directly or indirectly associated to neurotransmitters metabolism [31,32]. One of the most challenging scenarios is represented by the possible relationship between metabolic and brain disorders, considered generally unlinked but probably strictly connected [33]. An interesting example is given by TMAU, a metabolic disease characterized by fish odor emission due to the release of high TMA levels, previously accumulated in various body secretions like sweat, urine, blood and vaginal one [34]. While in the primary form of TMAU phenotype is mainly determined by genetic mutations in *FMO3* gene [35], in the secondary one the causes can be different: gut microbiome dysbiosis is one [1]. Patients affected by both primary and secondary forms of TMAU frequently show behavioral disturbs like social exclusion, depression, anxiety, sleep-wake cycle and humoral alterations, until to suicide attempt in extreme cases [36]. These psychological comorbidities, strictly linked to limbic system, represent the most controversial aspects of this pathology, because it is still unknown whether these disturbs are the consequences of social reactions to malodour or could depend on TMA-induced biochemical alterations of nervous system. To deepen this challenging point, we studied 12 patients affected by secondary TMAU, 7 of whom presenting a complex psychological or psychiatric clinical picture (namely called “cases”). All patients were subjected to microbiota analysis, highlighting differences in bacterial abundance and heterogeneity between cases and controls. The bacterial families that showed the most relevant differences in terms of relative abundances were, then, investigated for metabolic pathways. Very interestingly, the highest number of intermediates produced by gut microbiota is transported to central nervous system (CNS), especially to amygdala and hippocampus, through blood stream, even altering the blood brain barrier (BBB) permeability. Furthermore, the same metabolites can directly act on the autonomous nervous system, regulating synapses of vagus nerve in enteric nervous system (ENS) [37]. The most innovative aim of our retrospective comparison was the evaluation of the possible link between TMA and its precursors with metabolism of neurotransmitters involved in limbic system activity. Thus, we proposed a new potential scenario consisting in the explanation of the biochemical patterns involving behavioral disturbs in secondary TMAU affected patients.

**Figure 7:**
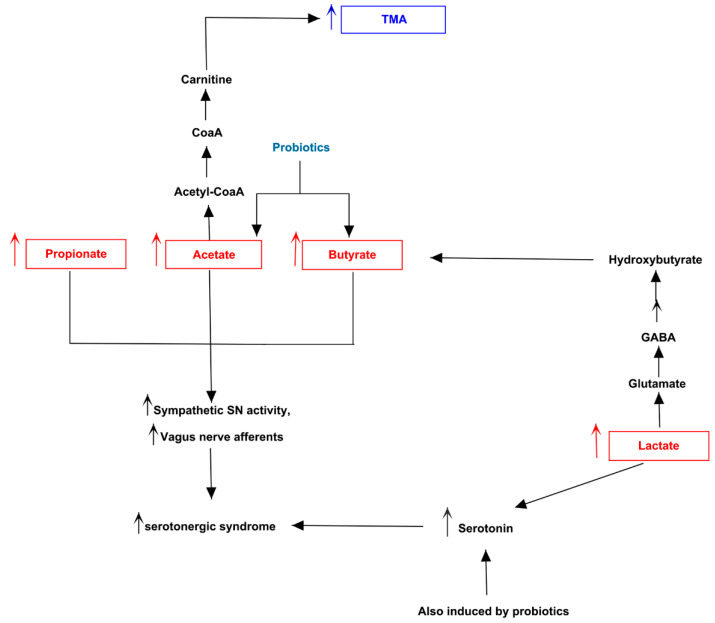
Biochemical pictures of TMAU patient 5. The panel represents how metabolites produced directly or indirectly by patient’s microbiota could influence the biosynthesis/release of neurotransmitters (in particular serotonin) and the production/accumulation of TMA.

Making a brief description of the cases, the patient 5 (Figure 7) potentially produced the lowest number of altered metabolites and showed an over-abundance of *Clostridiaceae* [38], related to high levels of main SCFAs (acetate, propionate and butyrate) and lactate. He manifested serotoninergic syndrome-like phenotype, especially obsessive-compulsive disturbs. This pathological condition is worsened by high lactate levels, which increase butyrate, by the assumption of antibiotics and by supplementation of probiotics consisting of *L. acidophilus*, *Bifidobacterium*, *L. rhamnosus*, *Streptococcus* and *L. paracasei*. Such bacterial families are known to increase the production of lactate, acetate, serotonin, GABA, also determining an accumulation of TMA.

**Figure 8:**
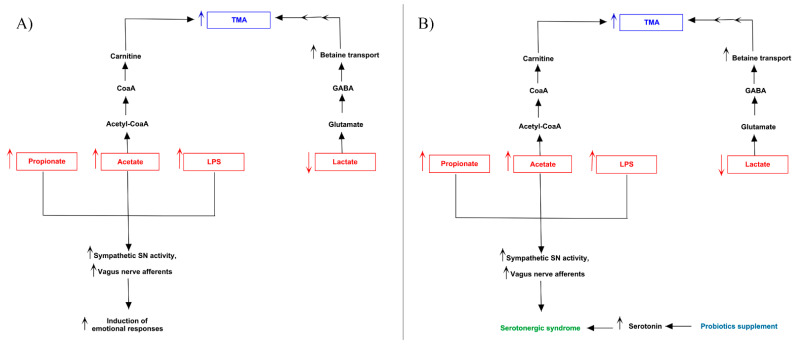
Biochemical pictures of TMAU patients 1 and 6. The panel represents how metabolites produced directly or indirectly by microbiota of patients 1 (**A**) and 6 (**B**) could influence the biosynthesis/release of neurotransmitters (in particular serotonin) and the production/accumulation of TMA.

Patients 1 (Figure 8A) and 6 (Figure 8B) showed an analogue serotoninergic syndrome-like symptomatology. The first patient presented an increase of gut *Enterococcaceae* and *Bacteroidaceae*, and a decrease of *Coriobacteriaceae* and *Ruminococcaceae*. The second one, instead, highlighted the highest number of differentially family’s composition, consisting of the increase of *Enterococcaceae*, *Erysipelotrichaceae*, *Rikenellaceae*, *Streptococcaceae* and *Coriobacteriaceae*, and the decrease of the only *Lachnospiraceae*. Dysbiosis of such bacteria families in both patients was related to augmented levels of acetate, propionate and LPS, while butyrate and lactate resulted decreased. The over-production of bacterial acetate can be involved into carnitine biosynthesis. The increasing of acetyl-Co, induced by acetate, can activate the carnitine biosynthesis by carnitine acetyl-transferase, thus triggering the accumulation of TMA. The known excitatory effects of lactate on neural metabolism can determinate an increase of both serotonin and glutamate, while provokes neurotoxicity in neural physiological environment [39]. Thus, low levels of lactate could reduce serotonin and glutamate, whose reduction might decrease GABA biosynthesis in central nervous system, mainly in hippocampus (https://www.proteinatlas.org/ENSG00000145692-BHMT/brain). This portion of limbic system expresses the betaine/GABA transporter BTG-1 [40] which, due to plasma low GABA concentration, might trigger the neuronal internalization of betaine. Betaine can be converted to TMA by betaine-homocysteine-S-methyltransferase (BHMT1) and a following decarboxylation. About serotonin, even if reduced lactate and butyrate levels could reduce it, the increase of acetate and propionate concentration can enhance its biosynthesis. Interestingly, the over-expression of the last two metabolites, together with LPS, could stimulate the afferent component of vagus nerve, inducing what is generally called “gut instincts” or visceral sensations. Such scenario can induce the brain to trigger emotional responses such as fear and anxiety, peculiar of patient 1. In patient 6, the augmented release of serotonin from enterochromaffin cells (ECCs) and the hyperactivation of vagus nerve can be linked to the probiotic supplementation of *L. helveticus* and *B. longum*, well known to increase serotonin and norepinephrine levels production in the hippocampus [41].

**Figure 9:**
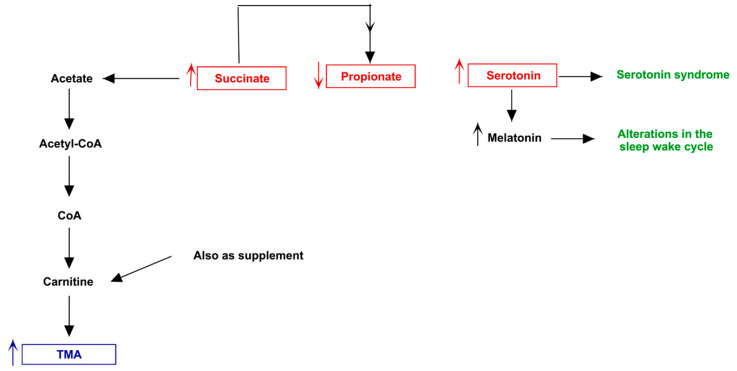
Biochemical pictures of TMAU patient 3. The panel represents how metabolites produced directly or indirectly by patient’s microbiota could influence the biosynthesis/release of neurotransmitters (in particular serotonin) and the production/accumulation of TMA.

A slightly different situation was evidenced by the patient 3 (Figure 9), who showed increased of *Enterococcaceae*, *Streptococcaceae* and *Prevotellaceae* relative abundance, linked to higher levels of succinate and serotonin and to low levels of propionate. We postulate that over-synthesis of succinate increases the levels of succinyl-CoA, which follows the biochemical pathway starting from succinic semialdehyde and determinates the final production of butyrate. The high levels of succinate and low levels of propionate probably produced by lactic acid mix fermentation, can determine an increase of acetate biosynthesis pathway that, as for patient 1, can imply an accumulation of TMA. Moreover, TMA levels could be increased by the supplement of L-carnitine, converted in TMA by bacterial carnitine oxidoreductase. The probable over-production of butyrate induced by succinate increases the serotonin biosynthesis by ECCs that, together with serotonin secreted by altered gut bacteria, can determine the phenotype typical of the serotoninergic syndrome. This condition reflects the major nervous-related symptoms shown by the patient (migraine, mood alteration, sense of marginalization and social phobia) [42]. Furthermore, the serotonin excess can increase levels of melatonin, explaining alteration of sleep-wake cycle of patient 3. 

**Figure 10:**
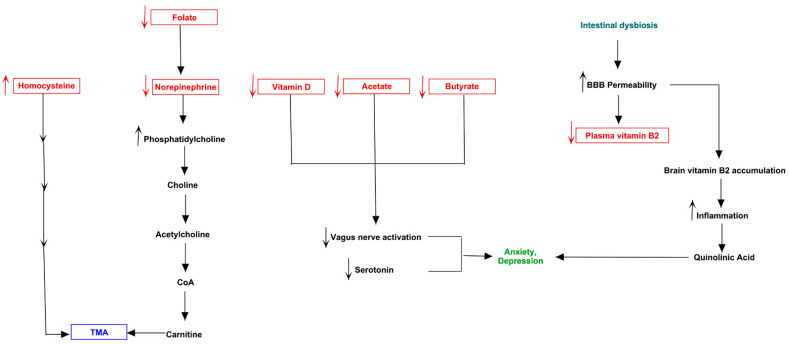
Biochemical pictures of TMAU patient 4. The panel represents how metabolites produced directly or indirectly by patient’s microbiota could influence the biosynthesis/release of neurotransmitters (in particular serotonin) and the production/accumulation of TMA.

A unique condition was evidenced in patient 4 (Figure 10). He presented a low of acetate, butyrate and Vitamin D levels, and increased concentration of propionate, suggesting a global reduction of vagus nerve activation and serotonin release, already determined by microbiota reduced abundances of *Streptococcaceae*. The low levels of folate characterizing the patient could impair the norepinephrine biosynthesis [43]. This event could shift the catalytic activity of PEMT from epinephrine biosynthesis towards phosphatidylcholine production, which could increase TMA levels via choline pathway. Furthermore, the high concentration of TMA could be also determined by elevated levels of homocysteine shown by the patient, through the reaction that transfer a methyl group from betaine to convert homocysteine to methionine, producing dimethylglycine (DMG) and, in subsequent step, TMA by decarboxylation. The most interesting metabolic pathway related to mood disorders was represented by low levels of plasmatic vitamin B2, which could be accumulated in nervous tissue following increased blood brain barrier (BBB) permeability. This permeability, indeed, is known to be caused by microbiota dysbiosis [44]. Moreover, this inflammatory scenario determined by altered microbiota could trigger the shifting of the tryptophan from serotonin pathway to degradation, producing kynurenine, which cross the BBB and, inside the nervous tissue, is converted into quinolinic acid [45]. This molecule is an antagonist of NMDA receptors and a non-competitive inhibitor of acetylcholine receptors, able to produce oxidative stress and neurotoxic effects, also inducing anxiety and depression, two behavioral alterations of patient 4.

**Figure 11:**
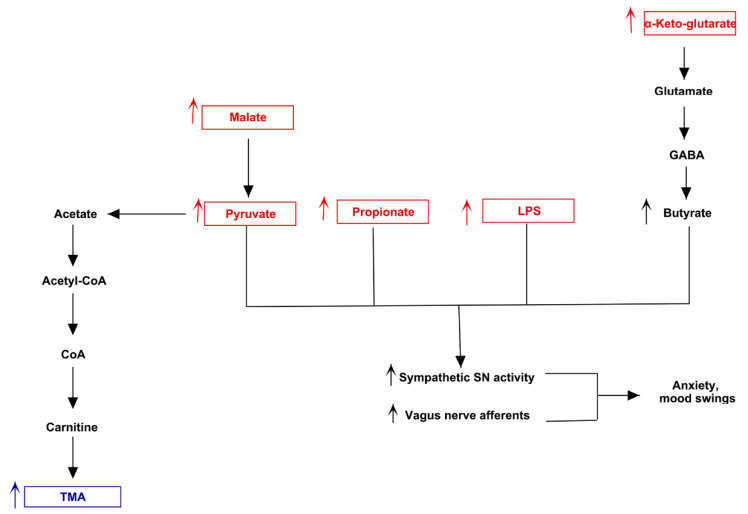
Biochemical pictures of TMAU patient 2. The panel represents how metabolites produced directly or indirectly by patient’s microbiota could influence the biosynthesis/release of neurotransmitters (in particular serotonin) and the production/accumulation of TMA.

The mixed acid fermentation is the biochemical pathway which produced the highest alteration in neural physiology-related metabolites in patient 2 (Figure 11). The increase of malate, mainly produced by *Oxalobacteraceae* [46], could stimulate the biosynthesis of pyruvate and, soon after, of acetyl-CoA. This metabolite is converted to acetyl phosphate, releasing CoA, with the synthesis of acetate in the final step. The CoA previously produced could enter in carnitine biosynthesis, leading to accumulation of TMA. Additionally, the high levels of alpha-ketoglutarate, together with low levels of lactate, could increase the succinic semi-aldehyde via GABA, determining the production of butyrate as fermentation product. Thus, the overall increase of main SCFAs, together with the elevated levels of propionate produced by altered microbiota, could favorite the ECC endogenous release of serotonin and the activation of the vagus nerve, along with LPS. Such scenario could explain the excess of anxiety and the uncontrolled emotional status.

**Figure 12:**
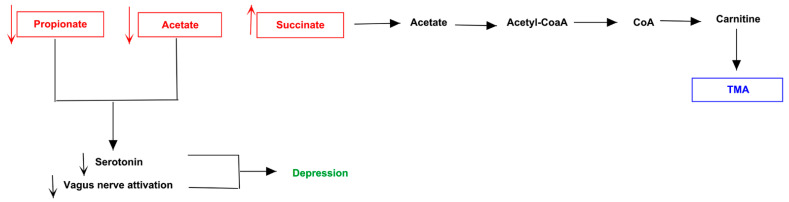
Biochemical pictures of TMAU patient 7. The panel represents how metabolites produced directly or indirectly by patient’s microbiota could influence the biosynthesis/release of neurotransmitters (in particular serotonin) and the production/accumulation of TMA.

A depressive phenotype was evidenced by patient 7 (Figure 12), who showed an increase of *Erysipelotrichaceae*, *Roseburia*, *Clostridiaceae* and *Prevotellaceae*, with a reduction of *Bacteroidaceae*. The alteration of these families could lead to a down-production of acetate and propionate, determining a global down-regulation of serotonin release and vagus nerve activation, characteristic of depression phenotype. In the meantime, the low levels of acetate could reduce the acetyl-CoA production, arresting the reaction which converts choline to acetylcholine. So, the accumulation of choline could augment TMA levels, leading to TMAU phenotype.

Based on both microbiota alteration evidences and host biochemical pathways, all analyzed cases showed relevant changes in production of behavioral disorder-related metabolites. In contrast controls here we considered highlighted different alterations in the same pathways. However, the intake of probiotic supplements balanced the pathological phenotype. This latter scenario characterizes controls 2c and 3c, who showed a different spectrum of metabolites. In addition, compensation of probiotics normalized the global concentration of the serotonin, as well as dopamine levels was balanced by *Enterococcus faecium* supplemented in subject 2c [47]. 

The metabolic picture of control 1c was characterized by a probable down-production of microbiota serotonin, due to decreased levels of several SCFAs and tryptophan. A possible compensation was provided by the human endogenous biosynthesis of serotonin, also enhanced by microbiota butyrate high levels.

An analogue condition was evidenced in control 4c, whose serotonin production induced by SCFAs could be balanced by reduction of vitamin D, which could decrease the neurotransmitter concentration. Moreover, the microbiota synthesis of dopamine might not exert positive effects on neurotransmission, due to the possible conversion of norepinephrine precursor to 6-hydroxydopamine (6-OHDA). Moreover, this could enhance the oxidative stress condition given by the high ROS levels detected in plasma.

Interestingly, the biochemical picture of control 5c highlighted how the increase of only *Prevotellaceae* and *Roseburia* might not be sufficient to determine a psychiatric phenotype. Probably the metabolites produced by both these bacteria are qualitative and quantitative not enough to exert a cytotoxic effect on nervous system. Thus, the integrity of psychic activities might be maintained or very little impaired.

All controls, considering the already discussed biochemical pathways analyzed in relation to cases, showed an accumulation of TMA.

### Limitations

Our results suggest that our hypothesis might be truly founded and they highly encourage to confirm them by further experiments. Therefore, we aimed to increase the statistical number of cases and controls, even if this pathology is enough rare to consider reliable our sample size. In order to improve the sample size in a useful way, we are also going to plan a more rigid clinical study, evaluating a stronger methodology. Regarding this, we are also going to improve the psychiatric anamnesis with more details, evaluate the biochemistry and molecular genetics of investigated metabolites, and realize several physiological essays in order to ensure the role of each metabolite in each considered pathway. Such approach could improve the group sampling, trying to avoid several biases caused by the lack of these data.

## 5. Conclusions

The relationship between gut microbiota and psychiatric disturbs is one of the most challenging topics involving researchers. The vagal nerve is the anatomical structure which permits the communication between the central nervous system (CNS) and enteric nervous system (ENS). Vagal afferent neurons express receptors for gut microbiota metabolites, such as serotonin, that can modulate nutrient metabolism. Furthermore, SCFAs, catecholamines, acetylcholine, the intermediates of mixed acid fermentation and TMAO are able to regulate metabolism through a microbiota-gut-liver axis. However, very little is known about the direct connection between metabolic diseases and mental disorders, involving common pathway in which the considered metabolites play an orchestral role. In our retrospective comparison, we laid the bases for further investigation about biochemical and biological link between secondary trimethylaminuria and psychiatric behaviors. We suppose that the mental disturbs affecting TMAU patients are probably not only related to social consequence of their metabolic disease but also to a physiopathological effect determined by TMA accumulation. The knowledge of this aspects might allow us to personally modulate each gut microbiota. Thus, the related microbiota-gut-brain axis may become a potential new strategy for improving prognosis of metabolic diseases and treat linked psychiatric disorders. 

## Figures and Tables

**Table 1:** Subject metabolic and behavioral features. TMAU patients with psychiatric symptomatology (1–7) and TMAU control patients without mental disturbs (1c–5c) were selected for our retrospective comparison, mainly in relationship with relevant differences of behavioral phenotypes.

ID	AGE	SEX	TMAU AGE of ONSET	DIET	ANTIBIOTIC MASSIVE USE	PROBIOTIC/FOOD SUPPLEMENTS	BEHAVIOR DISORDER	KIND OF BEHAVIOR DISORDER	OTHER
1	30	M	17	Chocolate, Eggs, Peas	NO	NO	YES	Anxiety, Fear, Suicidal instincts, Mood alteration	/
2	40	F	14	Fish, Vegetables	NO	NO	YES	Excessive emotionality, Anxiety	/
3	54	F	6	Dairy products, Meat, Fish	NO	L-carnitine, bromelain	YES	Migraine, Sleep disorders, Mood alteration, Sense of marginalization, Difficulties in social relations	/
4	45	F	7	Chocolate, Legumes, Eggs, Fish	YES	NO	YES	Chronic and rapid mental fatigue, Frequent headaches, Dizziness, Anxiety, Depression	Low levels of Folate, Plasmatic Vitamin B2 and D, Cu^2+^, Zn^2+^; High levels of PTH, homocysteine, Ca^2+^
5	44	M	34	Coffee, Tea, White Meat, Vegetables, Fish	YES	L. acidophilus, Bifidobacterium lactis, L. rhamnosus, Streptococcus thermophilus and L. Paracasei	YES	Obsessive-compulsive disorder, Sense of marginalization	/
6	36	F	9	Vegetables, Coffee, Eggs	YES	Zinc, selenium, folic acid, iron, inulin, magnesium, L. Helveticus, B. longum spp.longum, Vitamin B6, Vitamin B1 and Vitamin D	YES	Mood alteration, Sense of marginalization, Suicidal instincts	/
7	25	F	4	Fish, Eggs, Chocolate, Legumes	NO	NO	YES	Depression, Obsessive-compulsive disorder, Sense of persecution	/
1c	47	F	8	Gluten-free foods, Vegetables, Coffee	NO	NO	NO	NO	/
2c	26	M	10	Fish, Chocolate, Red meat, Coffee, Alcohol	NO	Bifidobacterium lactis, L. acidophilus, L. plantarum, L. paracasei; Streptococcus salivarius subsp. thermophilus, Bifidobacterium brevis, Lactobacillus delbrueckii subsp. bulgaricus, Enterococcus faecium.	NO	NO	/
3c	20	M	16	Gluten-free foods, Vegetables, Meat	NO	L. acidophilus, Bifidobacterium lactis, L. rhamnosus, Streptococcus thermophilus and L. Paracasei	NO	NO	/
4c	72	F	2	Gluten-free and Lactose-free foods, Fish	NO	NO	NO	NO	High ROS and Arachidonic Acid
5c	35	M	35	Red meat, Legumes, vegetables, Salmon	NO	NO	NO	NO	Use of alcohol

**Table 2:** Differentially represented bacterial families/genera in TMAU psychiatric cases and controls. Microbiota analysis of TMAU psychiatric cases and controls showed alterations (% relative abundance) for 16 families and 2 genera (Roseburia and Faecalibacterium). Over-representation are highlighted in red, down-representation in light blue. The normal range of % relative abundance is indicated between squared brackets.

ID	1	2	3	4	5	6	7	1c	2c	3c	4c	5c
*Enterobacteriaceae [0.1–1.1]*	0.85	1.08	0.45	0.1	0.74	0.15	0.15	0.02	0.01	0.1	2.8	0.05
*Oxalobacteraceae [0.0–0.0]*	0	0.05	0	0	0	0	0	0	0	0	0.08	0
*Enterococcaceae [0.0–0.0]*	0.02	0	0.02	0	0	0.68	0	0	0	0	0	0
*Erysipelotrichaceae [0.1–2.9]*	2.8	0.4	0.78	0.1	0.38	3.9	3.3	0.15	0.21	0.1	3.8	2.62
*Rikenellaceae [0.2–5.3]*	0.48	5.22	1.25	0.2	2.2	6.95	0.2	0.2	0.2	0.2	6.78	0.48
*Veilloneaceae [0.8–7.7]*	6.35	3.15	1.58	0.8	2.8	5.35	3.35	0.8	0.8	0.8	0.48	1.85
*Roseburia [0.0–0.9]*	0	0.15	0.25	0.85	0	0.04	1.03	3.09	4.4	0	1	1.53
*Streptococcaceae [0.1–1.8]*	0.28	0.22	3.48	0.01	0.15	2.62	0.15	0.1	0.1	0.03	0.32	0.08
*Clostridiaceae [0.1–1.4]*	0.28	1.45	1.25	287.8	134.1	0.28	1.6	0.1	0.1	0..23	0.32	0.18
*Lachnospiraceae [12.8–37.26]*	20.52	9.98	24.78	1.86	15.8	3.78	23.22	72.24	44.65	0.04	18.58	23.25
*Prevotellaceae [0.1–13.66]*	0.12	2.3	16.68	0.1	0.7	3.85	40.0	0.02	0.1	0.1	0.13	26.65
*Coriobacteriaceae [0.3–5.9]*	0.15	1.08	2.12	0.01	0.7	6.5	0.82	0.3	0.3	0.04	0.52	1.7
*Bacteroidaceae [3.2–35.36]*	55.62	17.5	9.98	3.2	9.2	25.38	1.4	3.2	3.2	3.2	14.58	9.45
*Ruminococcaceae [13.7–34.7]*	2.42	24.4	23.38	13.7	18.7	24.35	16.23	0.27	1.43	0.13	24.25	19.8
*Faecalibacterium [2.5–15.56]*	0	3.05	9.35	5.2	5.5	0.58	8.43	6.4	23.97	10.33	8.25	7.2
*Porphiromonodaceae [0.2–3.2]*	1.25	0.2	0.98	0.22	0.52	1.5	0.55	0.12	0.2	0.2	1.22	0.28
*Sutterellaceae [0.1–3.5]*	0.1	0.1	0.1	0.1	0.1	0.1	0.1	0.01	0.61	0.1	0.1	0.1
*Bifidobacteriaceae [0.1–7.96]*	4.38	1.82	0.38	0.39	3.55	3.88	0.1	0.1	0.003	0.11	0.1	1.05

**Table 3:** Metabolites produced by altered microbiotas related to neural metabolism. Differentially expressed families and genera of analyzed microbiotas showed a production of metabolites acting as intermediates of neural metabolism.

BACTERIA/METABOLITES	Lactate	Dopamine	Norepinephrine	Acetate	Serotonin	Succinate	Butyrate	Glycolate	Propionate	Pyruvate	α-ketoglutarate	LPS	Malate	Tryptophan	GABA
*Enterobacteriaceae*	X	X	X	X	X	X									X
*Oxalobacteraceae*	X						X	X		X	X		X		
*Enterococcaceae*	X				X							X			
*Erysipelotrichaceae*	X			X											
*Bifidobacteriaceae*	X			X											X
*Rikenellaceae*				X		X			X						
*Sutterellaceae*												X		X	
*Veilloneaceae*	X			X		X			X						
*Roseburia*	X			X			X		X						
*Ruminococcaceae*	X			X		X									
*Streptococcaceae*	X			X	X										
*Clostridiaceae*	X			X			X		X						
*Lachnospiraceae*	X			X		X	X								
*Prevotellaceae*				X		X			X						
*Coriobacteriaceae*	X			X											
*Bacteroidaceae*				X		X	X		X						X
*Faecalibacteriaceae*							X								
*Porphiromonodaceae*				X		X			X						

**Table 4:** Correspondence between altered microbiota families/genera and nervous-related metabolite levels. Differential abundances of bacterial families/genera leads to corresponding alterations of related metabolites acting as intermediate in neurophysiology. Considered metabolites only refer to microbiota biosynthesis, and they are retrieved from MACADAM database and literature. “↑” = over-production. “↓“ = down-production. “[empty space]” = no expression differences.

ID	1	2	3	4	5	6	7	1c	2c	3c	4c	5c
Acetate	↑			↓	↑	↑	↓	↓		↓	↓	↓
Lactate	↓	↓			↑	↓	↑		↓	↓	↑	
Succinate			↑			↑	↑	↓	↓	↓		
Dopamine									↓	↑	↑	
Norepinephrine				↓					↓	↑	↑	
Serotonin			↑	↓		↑			↓	↓	↑	
α-ketoglutarate		↑									↑	
Malate		↑									↑	
Pyruvate		↑									↑	
LPS	↑	↑								↓	↑	
Propionate	↑	↑	↓	↑	↑		↓	↓				
Butyrate	↓	↓		↓	↑	↓	↑	↑	↑	↑	↑	
Tryptophan								↓		↓		
GABA									↓			

## Supplementary Materials

The following are available online at https://www.mdpi.com/2075-4426/11/2/87/s1, Figure S1: Cladogram of most altered bacterial families in TMAU behavioral disordered cases.
