# Supplementary material for: Gut-Brain Axis Cross-Talk and Limbic Disorders as Biological Basis of Secondary TMAU
Source: J Pers Med. 2021 Jan 31;11(2):87. doi: 10.3390/jpm11020087 (PMC7912098; doi:10.3390/jpm11020087)
Supplement: Supplementary file 1 [file jpm-11-00087-s001.pdf]

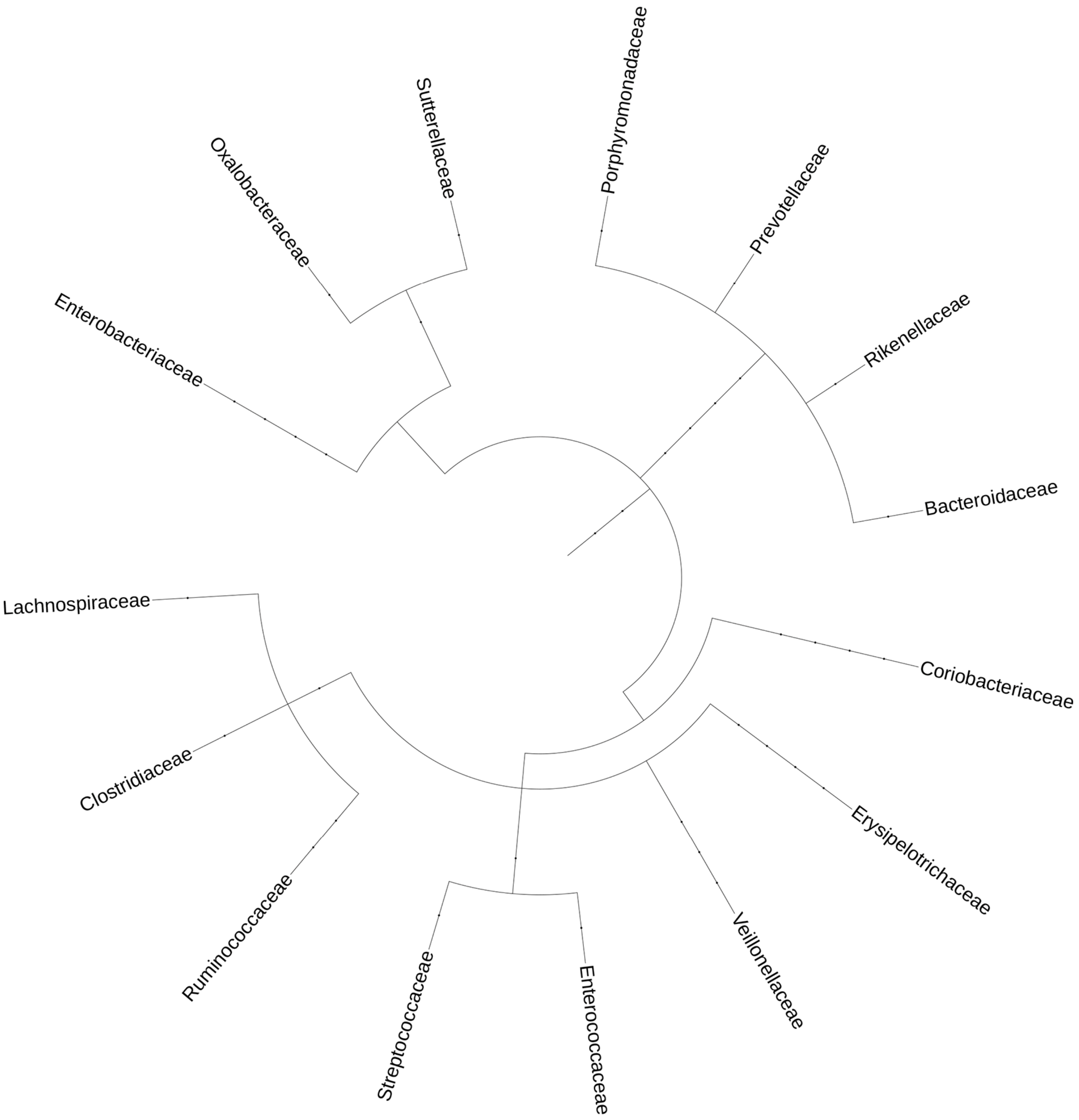

Figure S1. Cladogram of most altered bacterial families in TMAU behavioral disordered cases. All bacterial families which showed expression differences through microbiota of TMAU patients considered as cases resulted phylogenetically correlated each other.
